# Factors Associated with Active Trachoma in Alto Amazonas, Peru

**DOI:** 10.1080/09286586.2026.2702461

**Published:** 2026-10-28

**Authors:** John M. Nesemann, Noelia Morocho-Alburqueque, Alvaro Quincho-Lopez, Dionna M. Wittberg, Harvy A. Honorio-Morales, Salomón Durand, Martha Idalí Saboyá-Díaz, Ana Bakhtiari, Cristina Jimenez, Emma M. Harding-Esch, Sandra L. Talero, Marleny Muñoz, Anthony W. Solomon, Jeffrey D. Klausner, Jeremy D. Keenan, Andres G. Lescano

**Affiliations:** a Francis I Proctor Foundation, University of California San Francisco, San Francisco, California, USA; b Department of Ophthalmology, University of California San Francisco, San Francisco, California, USA; c Emerge, Emerging Diseases and Climate Change Research Unit, School of Public Health and Administration, Universidad Peruana Cayetano Heredia, Lima, Peru; d Piura Medical School, Piura, Peru; e Medical School, Universidad Nacional Mayor de San Marcos, Lima, Peru; f Componente de Salud Ocular y Prevención de la Ceguera, Ministerio de Salud, Lima, Perú; g Gerencia Regional de Salud Loreto, Iquitos, Perú; h Communicable Diseases Elimination Initiative, Pan American Health Organization, Washington DC, USA; i International Trachoma Initiative, Task Force for Global Health, Decatur, Georgia, USA; j Sightsavers, Haywards Heath, UK; k Clinical Research Department, London School of Hygiene & Tropical Medicine, London, UK; l Neglected, Tropical and Vector Borne Diseases Unit, Pan American Health Organization, Washington DC, USA; m Dirección de Epidemiología, Red de Salud Alto Amazonas, Yurimaguas, Perú; n Global Neglected Tropical Diseases Programme, World Health Organization, Geneva, Switzerland; o Division of Disease Prevention, Policy and Global Health, Department of Preventive Medicine, University of Southern California Keck School of Medicine, Los Angeles, California, USA

**Keywords:** Hygiene, risk factors, sanitation, trachoma, water

## Abstract

**Purpose:**

Factors associated with trachoma have been well characterized in Sub-Saharan Africa, including poverty, crowding, and poor access to water, sanitation, and hygiene. However, the relationship between these factors and trachoma may be different in non-African settings. The aim of this study was to determine the most important factors associated with active trachoma among children in an area of the Peruvian Amazon.

**Methods:**

Questionnaires and conjunctival photography were performed in a cross-sectional random sample of children aged 1–9 years from 21 communities in Alto Amazonas, Peru. Conjunctival photographs were graded by three trachoma experts for the presence of active trachoma.

**Results:**

Of 750 children with complete data, 229 had active trachoma. Factors most associated with active trachoma included living with a pre-school-aged sibling (adjusted prevalence ratio [PR] 1.61, 95%CI 1.23–2.11), having an unimproved water source for face-washing (PR 1.87, 95%CI 1.23–2.84), absence of a latrine (PR 1.68, 95%CI 1.04–2.70, comparing those with no latrine to those with access to an improved latrine), and household ownership of chickens (PR 1.58, 95%CI 1.13–2.21). Active trachoma was associated with lower scores on a hygiene index (PR 1.18 per unit decrement, 95%CI 1.04–1.33) and an asset-based wealth index (PR 1.14, 95%CI 1.02–1.28).

**Conclusions:**

The most important factors associated with active trachoma in this study were exposure to young children, poor access to water, sanitation, and hygiene, and indicators of poverty. These factors are similar to those of previous studies of trachoma in other parts of the world.

## Introduction

Trachoma, caused by repeated infections with ocular strains of *Chlamydia trachomatis*, remains the leading infectious cause of blindness worldwide. The World Health Organization (WHO) has targeted trachoma for elimination by 2030.^1-3^ Trachoma is a disease of poverty, and has consistently been found to be associated with indicators of poor water, sanitation, and hygiene, although the vast majority of studies have been done in Sub-Saharan Africa.^3,4^ Recent studies have found trachoma in the Amazon, where trachoma interventions are currently not widespread.^5,6^ The climate, geography, and culture of the Amazon is vastly different from settings in Africa where trachoma has been best characterized, and thus it is not self-evident that the factors traditionally associated with trachoma would be the same in the Amazon. We collected household-level exposure data as part of a population-based trachoma survey in the Alto Amazonas province of the Peruvian Amazon to assess factors associated with active trachoma. Our objective was to determine if indicators of poverty and poor access to water, sanitation, and hygiene – the factors typically associated with trachoma in Africa – were also associated with trachoma in Alto Amazonas.

## Methods

### Study design

The study was conducted in five of the six districts of Alto Amazonas province, Loreto region, Peru. The 2017 census estimated that indigenous people – primarily from the Shawi community – made up approximately 46% of the population of these 5 districts.^7^ The study design, described in detail elsewhere, adhered as closely as possible to recommendations from Tropical Data, which supports health ministries to conduct high-quality, standardized, trachoma prevalence surveys.^8,9^ In brief, after excluding Yurimaguas district and the capital city of each district given an expected low prevalence of trachoma in urban areas, 22 communities from Alto Amazonas province were selected using probability-proportional -to-size sampling. In each community, 30 households were randomly selected for participation. Field workers administered a risk factor survey to an adult at the household, with questions focused on demographic, hygiene, and socioeconomic factors (Table S1). The survey was developed with epidemiologists in Lima and Alto Amazonas and was based on questions included in Peru’s 2013 Demographic and Health Survey as well as in routine Tropical Data surveys. If consent was obtained, the upper eyelid of each eye of all children aged 1–9 years in the household was everted, examined for trachomatous inflammation – follicular (TF) and trachomatous inflammation – intense (TI) according to the WHO simplified grading system, and photographed with a smartphone camera (Xiaomi A2) with magnifying attachment (Corneal CellScope, University of California, Berkeley).^10,11^ A single dose of oral azithromycin or topical tetracycline was offered to all household members of participants graded as having TF or TI. Three masked graders who had been certified for trachoma grading by Tropical Data graded conjunctival photographs for the presence of TF and TI according to the WHO simplified grading system.^12^ The majority opinion between the three graders was used as the official trachoma grade for analyses.^11^ Photo-grades were used to assess trachoma in this study since photographs were auditable and provided the opportunity for multiple graders to provide a trachoma assessment.

### Statistical considerations

The sample size was chosen to provide sufficient precision for an estimate of TF prevalence at the level of rural Alto Amazonas.^8^ Hygiene variables were aggregated into a single hygiene index and socioeconomic variables into a single asset-based wealth index using a principal components analysis (PCA) of the non-standardized raw data, with eigenvectors derived from the correlation matrix.^13^ PCA results were visualized with plots depicting the percentage contribution of each variable to each principal component and with graphs of the first versus second components (Figure S1). The outcome for the present analysis was the presence of active trachoma, defined as TF or TI in at least one eye, and the exposures were the individual- or household-level variables. The prevalence ratio (PR) was estimated from mixed effects Poisson regression models that included (i) robust standard error estimates, (ii) community as a random intercept, and (iii) adjustment for age in years and district based on the theorized causal pathway (Figure S2). All analyses were performed using a complete-case approach. Regression analyses and PCA were performed with Stata 18; data management and graphing were performed in R version 4. The significance level was set to 0.05 for this hypothesis-generating study.

### Ethics

Approval was obtained from ethics committees at the University of California San Francisco, Universidad Peruana Cayetano Heredia, and Pan American Health Organization; from the regional health authorities in Loreto; and from community leaders. Approval was obtained from the London School of Hygiene & Tropical Medicine for routine Tropical Data support for the surveys (16105). Written informed consent was obtained from participants or their guardians, a copy of which was stored in the data collection software.

## Results

Fieldwork was performed between February 6, 2020 and March 14, 2020. Two communities could not be reached due to river conditions and were substituted with two nearby communities. Leaders of a single community refused to participate in the study, leaving 21 communities in the study population. A total of 388 of the 617 households visited during the study had at least one child aged 1–9 years, of which 382 (98%) consented to participate and completed a household survey. From these surveyed households, a total of 750 children aged 1–9 years were enumerated and photographed for trachoma (mean age 5.0 years, standard deviation 2.5; 367 [49%] female), with 222 (30%) classified as having TF in at least one eye and 51 (7%) classified as having TI in at least one eye.

The prevalence of active trachoma was higher in younger children (e.g., 129 of 328 [39%] children aged 1–4-years were graded as TF or TI compared with 100 of 422 [24%] children aged 5–9-years; crude PR 0.90 [95% CI 0.87–0.92] for each additional year of age up to 9 years) and varied by district, but was similar in males and females (Table 1). Household demographic characteristics are shown in Table 2. The household demographic factor most associated with active trachoma was having a pre-school-aged sibling (38% active trachoma in children with a pre-school sibling versus 21% in those without; adjusted PR 1.61, 95%CI 1.23–2.11). Hygiene characteristics are shown in Table 3. Active trachoma was most strongly associated with the source of water for face-washing (36% in children using water from an unimproved water source compared with 13% among those using an improved water source; adjusted PR 1.87, 95%CI 1.23–2.84) and the absence of a latrine (TF prevalence 38% in children with no latrine compared with 20% in those with an unimproved latrine and 23% in those with an improved latrine; adjusted PR 1.68, 95%CI 1.04–2.70, comparing the no latrine to improved latrine group). Children with lower scores on a hygiene index were more likely to have active trachoma (age- and district-adjusted PR 1.18 per unit decrement in the PCA-derived hygiene index, 95%CI 1.04–1.33; index captured 43% of the total variance; Figure 1). Asset-based socioeconomic characteristics are shown in Table 4. An upload error led to missing socioeconomic data for 83 children, although the age, gender, and district of the missing children was nearly identical to that of the complete cohort (see footnote in Table 4). The socioeconomic factor most strongly associated with TF was household ownership of chickens (32% for children whose household owned at least 1 chicken; 19% for children with no chickens; PR 1.58, 95%CI 1.13–2.21), followed by the amount of land farmed (38% if ≥3 hectares farmed; 29% if 2 hectares farmed; 33% if 1 hectare farmed; 27% if no land was farmed; PR 1.95, 95%CI 1.26–3.01, comparing the highest to lowest group). Children with lower scores on an asset-based socioeconomic index were more likely to have active trachoma (age- and district-adjusted PR 1.14 per unit decrement, 95%CI 1.02–1.28; index captured 17% of the total variance; Figure 1). Analyses were similar when conducted with TF as the outcome (Tables S2, S3, S4, and S5, and Figure S3).

## Discussion

We found that factors typically associated with clinical trachoma in sub-Saharan Africa were also associated with trachoma in the Peruvian Amazon, including young age, proximity to a pre-school child, indicators of poor personal hygiene, and an asset-based index of poverty.

This study’s finding that active trachoma was more common among younger children has been observed in many prior studies.^15^ The preponderance of inflammatory signs of trachoma among children is thought to be due to some combination of a naïve immune system and more frequent exposure to ocular *C. trachomatis* infections. Crowded conditions may enhance transmission, since a primary route of infection is thought to be via direct contact with ocular and nasal secretions.^16^ The association between crowding and trachoma would be greatest when many young children were living together, since these children would be more likely to be infected. The association between active trachoma and (i) younger age, (ii) living with a pre-school-aged child, and (iii) large household size in the present study suggests that trachoma in the Amazon behaves similarly to trachoma elsewhere in the world.

The association between poor hygiene and TF found in this study is consistent with a large body of observational research conducted in a variety of settings throughout the world, although mostly in Africa.^4^ The importance of poor hygiene in the present study was not necessarily to be expected given the starkly different climate and geography of the Amazon compared to sub-Saharan Africa. For example, trachoma has typically been associated with inadequate access to water for face-washing, which should be less of an issue for residents of communities situated on a river. The association between trachoma and open defecation is thought to be mediated by the fly *Musca sorbens*, which can act as a mechanical vector for ocular *C. trachomatis* and breeds preferentially in human feces.^17,18^ However, *M sorbens* is not present in the Americas. While one study done in Brazil found weak associations between other species of synanthropic flies and trachoma, little evidence exists to suggest that flies are a major contributor to the transmission of trachoma in the Americas.^19^ Other studies on factors associated with trachoma specifically in the Amazonian context are limited, although studies done in Colombia and Peru found that children with unclean faces were more likely to have TF, and the Colombian survey also found a significant association between TF and open defecation near the household.^5,20^

In addition to individual- and household-level associations, certain ecological factors have been found to be associated with trachoma, such as dry climate, lower temperature, low altitude, remote and rural setting, and poor access to health services, schools, and water and sanitation.^21^ Although the Amazon would not be considered dry or cool, the study communities were certainly remote and largely agrarian. Indeed, several of the socioeconomic factors associated with active trachoma – for example, hectares owned and chicken ownership – would be consistent with a higher prevalence of trachoma among rural and agrarian households. Study communities also had relatively poor access to health services due to their remoteness, and limited water and sanitation infrastructure. Thus, it is likely that the Amazonian communities in the present study share commonalities with other trachoma-endemic communities elsewhere in the world.

WHO recommends the “SAFE strategy” for trachoma elimination: Surgery for trachomatous trichiasis, Antibiotics to clear ocular *C. trachomatis*, Facial cleanliness to reduce transmission of ocular and nasal secretions, and Environmental improvement to facilitate access to water and sanitation.^22^ The present study’s finding that indicators of poor hygiene were associated with trachoma in the Amazon, just as they are in Africa, could suggest a common causal pathway, and offer support for adopting the SAFE strategy in the Amazon. However, it is impossible to study causality in a cross-sectional observational study: it is also plausible that poor hygiene is simply a marker of poverty and does not lie on the causal pathway for developing active trachoma. More evidence from randomized trials is needed to better characterize causality, especially since existing randomized trials that have implemented hygiene interventions for trachoma have not offered conclusive evidence of benefit.^23-27^

Indices of hygiene and socioeconomic status were derived from PCA under the rationale that the different variables were likely correlated with each other. The hygiene index accounted for approximately 43% of the total variance of the hygiene variables. Although right-skewed with some evidence of truncation and clustering (e.g., relatively little variability between households), the distribution of values of the hygiene index was heterogeneous enough to detect a significant association with active trachoma. In contrast, the asset-based wealth index was a weaker indicator in this study, accounting for only 17% of the total variance of the wealth-based variables. While the distribution of wealth index values did not exhibit clustering or truncation, the association between this indicator and active trachoma was not as strong as that of the hygiene indicator. The weaker association between the wealth index and active trachoma may be due to an insensitive questionnaire that focused on assets or may simply reflect a relatively homogenous population for which assets were similar.

This study has limitations. Unlike other recent population-based trachoma surveys that have trained field graders, trachoma was assessed in the present study from conjunctival photographs. While this differs from usual practice, smartphone imaging with the CellScope (i.e., the attachment used in the present study) has shown acceptable agreement with field grades for TF and TI.^28^ Moreover, the use of conjunctival photography allowed photo-graders certified by Tropical Data to reach a majority consensus, increasing confidence and generalizability of the results and allowing the grades to be audited. These advantages were especially important for the present study, since field workers had no prior experience grading trachoma and because the nature of fieldwork on the river prevented active monitoring and remediation of trachoma graders. Data on asset-based wealth indicators were missing for approximately 10% of the sample due to an upload error. Although there was no evidence that children with missing data were systematically different from those without (Table 4 footnote) it is impossible to know whether analyses of a complete dataset would have led to different conclusions. The factors assessed in the present study are a small subset of all possible factors associated with TF, and a different set of questions may have given different results. Although it is likely that the findings are generalizable to other trachoma-endemic settings in the Amazon River basin, the study’s generalizability outside the specific study area is unclear.

In summary, we found a similar set of factors associated with trachoma in the Peruvian Amazon as has been observed in various settings in Africa, despite the different geographic and climatic setting. Further studies are indicated to assess whether interventions addressing these factors in an Amazonian setting are effective for reducing active trachoma.

## Supplementary Material

Supp 1

Supplemental data for this article can be accessed online at https://doi.org/10.1080/09286586.2026.2702461.

## Figures and Tables

**Figure 1.**
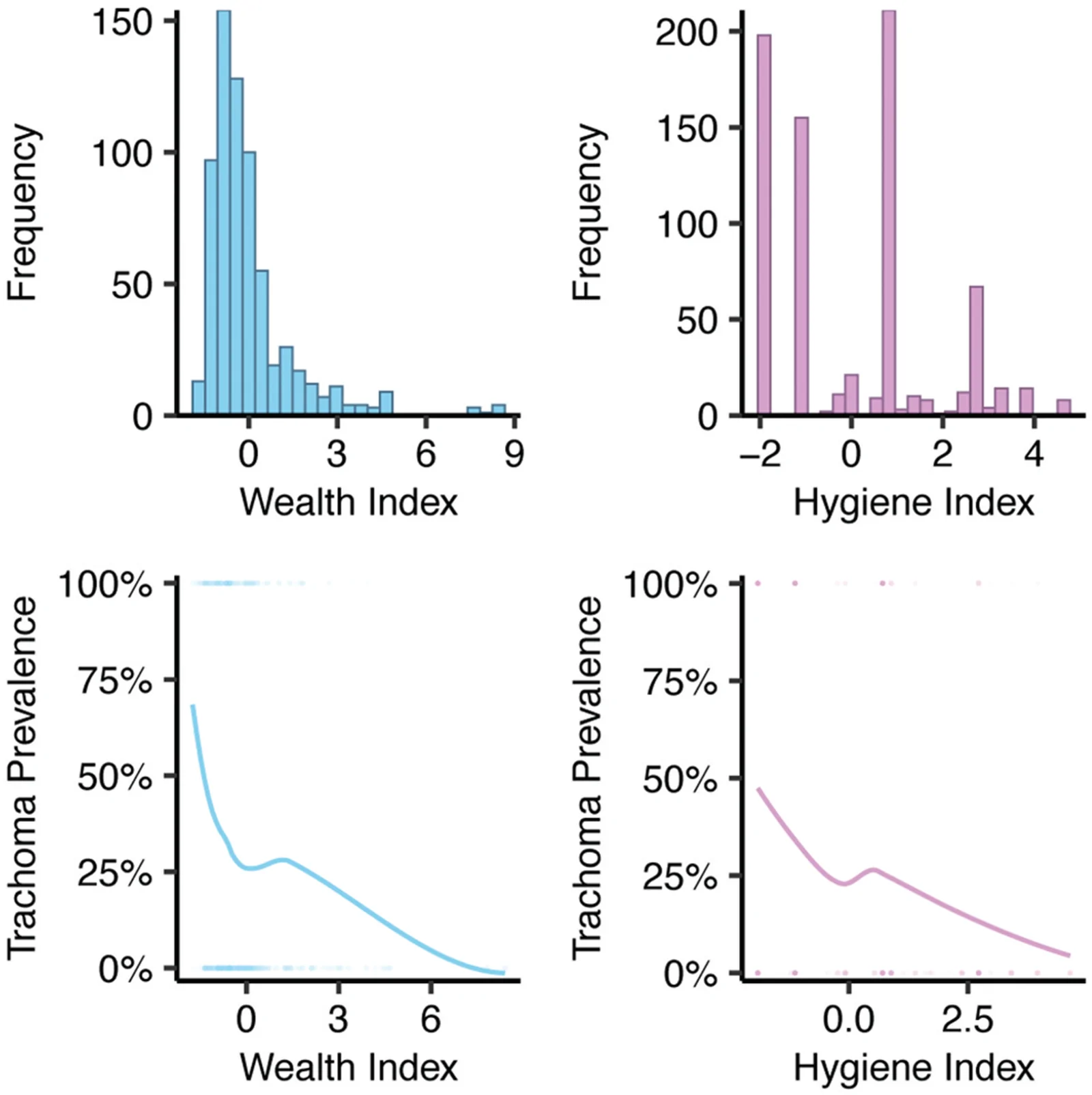
Relationship between wealth and hygiene indices with trachoma. The top panels show histograms of the wealth index (left) and hygiene index (right), as calculated from a principal components analysis. The bottom panels are loess curves depicting the relationship of the wealth index (left) and hygiene index (right) and the prevalence of trachomatous inflammation – follicular (TF).

**Table 1.** Associations between individual-level demographic variables and active trachoma among children aged 1–9 years in Alto Amazonas, Peru. Active trachoma was defined as the presence of trachomatous inflammation – follicular (TF) or trachomatous inflammation – intense (TI).

Variable	Prevalence of active trachoma	Prevalence Ratio (95%CI)	P-value
**Age, years**			
1	29 / 78 (37%)	Ref	
2	38 / 84 (45%)	1.25 (0.95–1.64)	
3	25 / 79 (32%)	0.95 (0.68–1.32)	
4	37 / 87 (43%)	1.16 (0.90–1.49)	
5	27 / 83 (33%)	1.01 (0.73–1.40)	<0.001
6	28 / 87 (32%)	0.93 (0.62–1.37)	
7	19 / 82 (23%)	0.67 (0.45–1.00)	
8	15 / 100 (15%)	0.44 (0.30–0.66)	
9	11 / 70 (16%)	0.43 (0.27–0.68)	
**Gender**			
Female	113 / 367 (31%)	Ref	
Male	116 / 383 (30%)	1.11 (0.94–1.30)	0.22
**District**			
Balsapuerto	126 / 338 (37%)	Ref	
Jeberos	53 / 131 (40%)	0.89 (0.40–1.95)	
Lagunas	17 / 96 (18%)	0.48 (0.23–1.02)	0.10
Santa Cruz	11 / 81 (14%)	0.27 (0.07–0.96)	
Shucushyacu	22 / 104 (21%)	0.36 (0.06–2.13)	

**Table 2.** Associations between household-level demographic variables and active trachoma among children aged 1–9 years in Alto Amazonas, Peru. Active trachoma was defined as the presence of trachomatous inflammation – follicular (TF) or trachomatous inflammation – intense (TI).

Variable	Prevalence of active trachoma	Prevalence Ratio (95%CI)^a^	P-value
**Gender of head of household**			
Male	192 / 614 (31%)	Ref	
Female	37 / 136 (27%)	1.25 (1.04–1.51)	0.02
**People per household**			
≤6 people	134 / 510 (26%)	Ref	
>6 people	95 / 240 (40%)	1.22 (1.04–1.43)	0.02
**Sibling aged ≤ 9y in household**			
No	22 / 123 (18%)	Ref	
Yes	207 / 627 (33%)	1.53 (1.10–2.13)	0.01
**Sibling aged ≤ 4y in household**			
No	64 / 311 (21%)	Ref	
Yes	165 / 439 (38%)	1.61 (1.23–2.11)	<0.001

a Adjusted for age in years and 5-level district variable.

**Table 3.** Associations between hygiene variables and active trachoma among children aged 1–9 years in Alto Amazonas, Peru. Active trachoma was defined as the presence of trachomatous inflammation – follicular (TF) or trachomatous inflammation – intense (TI).

Hygiene characteristic^a^	Prevalence of active trachoma	Prevalence Ratio (95%CI)^b^	P-value
**Drinking water source quality** ^c^			
Improved	7 / 70 (10%)	Ref	
Unimproved	222 / 680 (33%)	2.59 (0.90–7.47)	0.08
**Round trip time to get drinking water**			
Yard	3 / 32 (9%)	Ref	
<30 mins	223 / 709 (33%)	1.34 (0.52–3.46)	0.82
≥30 mins	3 / 9 (33%)	1.40 (0.47–4.19)	
**Face washing water source quality** ^d^			
Improved	23 / 177 (13%)	Ref	
Unimproved	206 / 573 (36%)	1.87 (1.23–2.84)	0.003
**Round trip time to get washing water**			
Yard	21 / 153 (14%)	Ref	
<30 mins	188 / 518 (36%)	1.51 (0.93–2.47)	0.13
≥30 mins	20 / 79 (25%)	1.03 (0.47–2.30)	
**Household latrine** ^b^			
Improved latrine	20 / 87 (23%)	Ref	
Unimproved latrine	50 / 246 (20%)	1.34 (0.80–2.24)	0.07
None	159 / 417 (38%)	1.68 (1.04–2.70)	
**Defecation location**			
Private latrine	68 / 313 (22%)	Ref	
Outside / open defecation	158 / 415 (38%)	1.37 (1.03–1.83)	0.07
Shared latrine	3 / 22 (14%)	0.82 (0.32–2.07)	
**Child feces disposal**			
No child under 3	39 / 240 (16%)	Ref	
Disposed of in latrine/trash	64 / 218 (29%)	1.41 (0.91–2.18)	0.12
Buried	26 / 57 (46%)	1.59 (0.94–2.70)	
Left in open	100 / 235 (43%)	1.52 (1.08–2.14)	

a Each variable was included in the principal components analysis generating a hygiene index. Multi-level categorical variables were dichotomized as follows: time to get water (yard vs. farther), household latrine (presence vs absence), defecation location (latrine vs open), child feces disposal (first three levels vs left in open).

b Adjusted for age in years and district.

c Dichotomized according to the WHO / UNICEF Joint Monitoring Program recommendations. Improved: protected dug well, piped to compound, protected spring, public tap/stand pipe. Unimproved: surface water, unprotected dug well, unprotected spring.^14^

d Classified according to the WHO / UNICEF Joint Monitoring Program recommendations. Improved: flush/pour to pit latrine, flush/pour to septic tank, flush/pour to unknown place, pit latrine with slab, ventilated improved pit latrine, composting toilet. Unimproved: pit latrine without slab/open pit, no facilities or bush or field or surface water, hanging toilet/hanging latrine, flush/pour to open drains, unable to observe private latrine.^14^

**Table 4.** Associations between socioeconomic variables and active trachoma among children aged 1–9 years in Alto Amazonas, Peru. Active trachoma was defined as the presence of trachomatous inflammation – follicular (TF) or trachomatous inflammation – intense (TI).

Characteristic^a^	TF Prevalence^b^	Prevalence Ratio (95%CI)^c^	P-value
**Head of household occupation**			
Non-agriculture	13 / 74 (18%)	Ref	
Agriculture	177 / 557 (32%)	1.49 (0.84–2.62)	0.37
Unemployed or unpaid labor	16 / 36 (44%)	1.43 (0.83–2.47)	
**Hectares farmed**			
0	35 / 128 (27%)	Ref	
1	81 / 246 (33%)	1.27 (0.96–1.68)	0.02
2	66 / 230 (29%)	1.30 (0.99–1.70)	
≥3	24 / 63 (38%)	1.95 (1.26–3.01)	
**Chickens**			
No	16 / 78 (19%)	Ref	
Yes	190 / 589 (32%)	1.58 (1.13–2.21)	0.008
**Pigs**			
No	192 / 631 (30%)	Ref	
Yes	14 / 36 (39%)	1.31 (0.77–2.22)	0.32
**Cattle**			
No	199 / 648 (31%)	Ref	
Yes	7 / 19 (37%)	1.18 (0.57–2.44)	0.65
**Number of rooms**			
One	29 / 70 (41%)	0.96 (0.80–1.16)	0.66
Two or more	177 / 597 (30%)	Ref	
**Floor type**			
Cement / wood	84 / 377 (22%)	Ref	
Dirt	122 / 290 (42%)	1.20 (0.87–1.64)	0.26
**Window(s)**			
Yes	38 / 186 (20%)	Ref	
No	168 / 481 (35%)	1.27 (0.94–1.71)	0.11
**Cooking fuel**			
Electricity / natural gas	0 / 10 (0%)	Ref	
Wood	206 / 657 (31%)	8.54 (0.47–153)^d^	0.15
**Wardrobe**			
Yes	1 / 20 (5%)	Ref	
No	205 / 647 (32%)	4.50 (0.54–37.4)	0.17
**Mobile phone**			
Yes	16 / 80 (20%)	Ref	
No	190 / 587 (32%)	1.12 (0.59–2.14)	0.72
**Radio**			
Yes	48 / 163 (29%)	Ref	
No	158 / 504 (31%)	1.06 (0.84–1.35)	0.63
**Hammock**			
Yes	138 / 436 (32%)	Ref	
No	68 / 231 (29%)	0.86 (0.64–1.16)	0.32

a Each variable was included in the principal components analysis as listed here except for the following variables, for which continuous values were used: hectares farmed (mean 1.4, standard deviation [SD] 1.2), number of chickens (mean 11.4, SD 7.7), and number of rooms (mean 2.3, SD 0.7).

b Data on socioeconomic variables missing for 83 children across all communities due to an upload error. Characteristics of the 83 missing children were similar to the complete cohort: mean age 5.0, SD 2.5; 327 (49%) female; 200 (30%) with TF.

c Adjusted for age and district.

d Firth logistic regression, ignoring community-level clustering.
