# Supplementary material for: Factors Associated with Active Trachoma in Alto Amazonas, Peru
Source: Ophthalmic Epidemiol. Author manuscript; Available in PMC 2026 Oct 29. (PMC13718684; doi:10.1080/09286586.2026.2702461)
Supplement: Supplementary file 1 [file NIHMS2197339-supplement-Supp_1.docx]

Supplemental Appendix

Table S1. Survey questions…………………………………………………………………………………………………………….2

Table S2. Associations between individual-level demographic variables and trachomatous inflammation—follicular……….4

Table S3. Associations between household-level demographic variables and trachomatous inflammation—follicular……...5

Table S4. Associations between hygiene variables and trachomatous inflammation—follicular……………………………….6

Table S5. Associations between socioeconomic variables and trachomatous inflammation—follicular……………………....7

Figure S1. Directed acyclic graph depicting putative causal pathway between poverty and trachoma….…..………………..8

Figure S2. Visualization of principal components analysis………………………………………………………..………………..9

Figure S3. Relationship between wealth and hygiene indices with trachomatous inflammation—follicular………………....10

**Table S1.** Survey questions.

| In the dry season, what is the main source of drinking-water for members of your household? | 01 | Piped water into dwelling |
| --- | --- | --- |
|  | 02 | Piped water to compound/yard/plot |
|  | 12 | Piped water to neighbour |
|  | 03 | Public tap/standpipe |
|  | 04 | Tubewell/borehole |
|  | 05 | Protected dug well |
|  | 06 | Unprotected dug well |
|  | 07 | Protected spring |
|  | 08 | Unprotected spring |
|  | 09 | Rainwater collection |
|  | 10 | Delivered water (water vendor) |
|  | 13 | Water kiosk |
|  | 14 | Packaged water (bottled water, sachet water) |
|  | 11 | Surface water (e.g. river, dam, lake, pond, stream, canal, irrigation channel) |
|  | 99 | Other (specify next) |
| Please specify drink source | text |  |
| How long does it take to go there, get water, and come back? | integer |  |
|  | 000 | In the yard/plot |
|  | 999 | Don’t know |
| In the dry season, what is the main source of water used by your household for washing faces? | 01 | Piped water into dwelling |
|  | 02 | Piped water to compound/yard/plot |
|  | 12 | Piped water to neighbour |
|  | 03 | Public tap/standpipe |
|  | 04 | Tubewell/borehole |
|  | 05 | Protected dug well |
|  | 06 | Unprotected dug well |
|  | 07 | Protected spring |
|  | 08 | Unprotected spring |
|  | 09 | Rainwater collection |
|  | 10 | Delivered water (water vendor) |
|  | 13 | Water kiosk |
|  | 14 | Packaged water (bottled water, sachet water) |
|  | 11 | Surface water (e.g. river, dam, lake, pond, stream, canal, irrigation channel) |
|  | 99 | Other (specify next) |
| Please specify wash water source | text |  |
| How long does it take to go there, get wash water, and come back? | integer |  |
|  | 000 | In the yard/plot |
|  | 888 | All face washing done at water source |
|  | 999 | Don’t know |
| Where do you and other adults in the household usually defecate? | 01 | Shared or public latrine |
|  | 02 | Private latrine |
|  | 03 | No structure, outside somewhere |
|  | 99 | Other |
| Ask latrine/toilet type | 01 | Flush/pour flush to piped sewer system |
|  | 02 | Flush/pour flush to septic tank |
|  | 03 | Flush/pour flush to pit latrine |
|  | 04 | Flush/pour flush to open drains |
|  | 05 | Flush/pour flush to unknown place |
|  | 06 | Ventilated improved pit latrine (VIP) |
|  | 07 | Pit latrine with slab |
|  | 08 | Pit latrine without slab/open pit |
|  | 09 | Composting toilet |
|  | 10 | Bucket |
|  | 11 | Hanging toilet/hanging latrine |
|  | 12 | No facilities or bush or field or surface water |
|  | 13 | Container based sanitation |
|  | 14 | Not able to access (only select if unable to observe private latrine) |
|  | 99 | Other (specify next) |
| Please specify latrine type | text |  |
| If you have one or more children under 3 years of age resident in the household, the last time the youngest child passed faeces, what was done to dispose of the faeces? | 001 | Child used toilet/latrine |
|  | 002 | Put into toilet/latrine |
|  | 003 | Put into drain or ditch |
|  | 004 | Thrown into garbage |
|  | 005 | Buried |
|  | 006 | Left in the open |
|  | 007 | Don’t know |
|  | 009 | Other |
|  | 999 | There is no child under 3 years of age resident in the household |
| Occupation of head of household | 01 | Agricultural worker |
|  | 02 | Non-agricultural labor |
|  | 03 | Self-employed |
|  | 04 | Intellectual work |
|  | 05 | Fisherman |
|  | 06 | Unpaid housework |
|  | 07 | Paid housework |
|  | 08 | Unemployed |
| How many hectares of agricultural land do you own? | integer |  |
| How many guinea pigs do you own? | integer |  |
| How many pigs do you own? | integer |  |
| How many cows do you own? | integer |  |
| How many chickens do you own? | integer |  |
| Do you own the following? (multiselect) | Yes/no | Wardrobe |
|  | Yes/no | Blender |
|  | Yes/no | Motorcycle |
|  | Yes/no | Mobile phone |
|  | Yes/no | Windows |
|  | Yes/no | Radio |
|  | Yes/no | Solar panel |
|  | Yes/no | Hammock |
| What type of windows do you have in your household? | 00 | Glass |
|  | 01 | Wooden |
|  | 02 | Curtain |
|  | 03 | Screen |
| What type of floor do you use in the house? | 01 | Cement or brick |
|  | 02 | Granite, ceramic laminate |
|  | 03 | Asphalt or vinyl sheet |
|  | 04 | Wood |
|  | 05 | Dirt or sand |
|  | 06 | Other |
| If other floor type, please specify | text |  |
| What type of cooking fuel do you use in the household? | 01 | Electricity |
|  | 02 | Natural gas |
|  | 03 | Liquid gas (GLP) |
|  | 04 | Wood |
|  | 05 | Do not cook |
| How many rooms are in the household? | 01 | No divisions inside of housing / single space |
|  | 02 | Two rooms |
|  | 03 | Three rooms |
|  | 04 | Four or more rooms |

**Table S2.** Associations between individual-level demographic variables and trachomatous inflammation—follicular (TF) among children aged 1-9 years in Alto Amazonas, Peru.

| Variable | TF Prevalence | Prevalence Ratio (95%CI) | P-value |
| --- | --- | --- | --- |
| **Age (years)** |  |  |  |
| 1 | 26 / 78 (33%) | Ref |  |
| 2 | 37 / 84 (44%) | 1.33 (1.04–1.71) |  |
| 3 | 25 / 79 (32%) | 1.04 (0.76–1.42) |  |
| 4 | 36 / 87 (41%) | 1.24 (0.98–1.56) |  |
| 5 | 27 / 83 (33%) | 1.11 (0.80–1.54) | <0.001 |
| 6 | 27 / 87 (31%) | 0.99 (0.67–1.45) |  |
| 7 | 19 / 82 (23%) | 0.74 (0.49–1.11) |  |
| 8 | 15 / 100 (15%) | 0.48 (0.34–0.69) |  |
| 9 | 10 / 70 (14%) | 0.43 (0.26–0.72) |  |
| **Gender** |  |  |  |
| Female | 109 / 367 (30%) | Ref |  |
| Male | 113 / 383 (30%) | 1.12 (0.95–1.31 | 0.19 |
| **District** |  |  |  |
| Balsapuerto | 126 / 338 (37%) | Ref |  |
| Jeberos | 49 / 131 (37%) | 0.73 (0.25–2.15) |  |
| Lagunas | 17 / 96 (18%) | 0.48 (0.23–1.02) | 0.09 |
| Santa Cruz | 11 / 81 (14%) | 0.26 (0.07–0.96) |  |
| Shucushyacu | 19 / 104 (18%) | 0.32 (0.06–1.80) |  |

**Table S3.** Associations between household-level demographic variables and trachomatous inflammation—follicular (TF) among children aged 1-9 years in Alto Amazonas, Peru.

| Variable | TF Prevalence | Prevalence Ratio (95%CI)^a^ | P-value |
| --- | --- | --- | --- |
| **Gender of head of household** |  |  |  |
| Male | 185 / 614 (30%) | Ref |  |
| Female | 37 / 136 (27%) | 1.27 (1.05–1.54) | 0.01 |
| **People per household** |  |  |  |
| ≤ 6 people | 129 / 510 (25%) | Ref |  |
| > 6 people | 93 / 240 (39%) | 1.24 (1.03–1.50) | 0.02 |
| **Sibling aged ≤ 9y in household** |  |  |  |
| No | 22 / 123 (18%) | Ref |  |
| Yes | 200 / 627 (32%) | 1.48 (1.07–2.05) | 0.02 |
| **Sibling aged ≤ 4y in household** |  |  |  |
| No | 63 / 311 (20%) | Ref |  |
| Yes | 159 / 439 (36%) | 1.58 (1.21–2.05) | <0.001 |
| ^a^ Adjusted for age in years and 5-level district variable | | | |

**Table S4.** Associations between hygiene variables and trachomatous inflammation—follicular (TF) among children aged 1-9 years in Alto Amazonas, Peru.

| **Hygiene characteristic** | TF Prevalence | Prevalence Ratio (95%CI)^a^ | P-value |
| --- | --- | --- | --- |
| **Drinking water source quality^b^** |  |  |  |
| Improved | 6 / 70 (9%) | Ref |  |
| Unimproved | 216 / 680 (32%) | 3.12 (0.92–10.6) | 0.07 |
| **Round trip time to get drinking water** |  |  |  |
| Yard | 3 / 32 (9%) | Ref |  |
| <30 mins | 216 / 709 (30%) | 1.24 (0.48–3.19) | 0.89 |
| ≥30 mins | 3 / 9 (33%) | 1.30 (0.44–3.89) |  |
| **Face washing water source quality^c^** |  |  |  |
| Improved | 23 / 177 (13%) | Ref |  |
| Unimproved | 199 / 573 (35%) | 1.84 (1.17–2.87) | 0.008 |
| **Round trip time to get washing water** |  |  |  |
| Yard | 21 / 153 (14%) | Ref |  |
| <30 mins | 182 / 518 (35%) | 1.44 (0.82–2.53) | 0.19 |
| ≥30 mins | 19 / 79 (24%) | 0.96 (0.42–2.21) |  |
| **Household latrine^b^** |  |  |  |
| Improved latrine | 18 / 87 (21%) | Ref |  |
| Unimproved latrine | 50 / 246 (20%) | 1.45 (0.89–2.37) | 0.03 |
| None | 154 / 417 (37%) | 1.74 (1.15–2.64) |  |
| **Defecation location** |  |  |  |
| Private latrine | 66 / 313 (21%) | Ref |  |
| Outside / open defecation | 153 / 415 (37%) | 1.34 (1.01–1.79) | 0.08 |
| Shared latrine | 3 / 22 (14%) | 0.85 (0.34–2.14) |  |
| **Child feces disposal** |  |  |  |
| No child under 3 | 38 / 240 (16%) | Ref |  |
| Disposed of in latrine/trash | 60 / 218 (28%) | 1.38 (0.89–2.14) | 0.08 |
| Buried | 26 / 57 (46%) | 1.67 (0.97–2.87) |  |
| Left in open | 98 / 235 (42%) | 1.53 (1.09–2.15) |  |
| ^a^ Adjusted for age in years and district.  ^b^ Dichotomized according to the WHO / UNICEF Joint Monitoring Program recommendations. Improved: protected dug well, piped to compound, protected spring, public tap/stand pipe. Unimproved: surface water, unprotected dug well, unprotected spring.  ^c^ Classified according to the WHO / UNICEF Joint Monitoring Program recommendations. Improved: flush/pour to pit latrine, flush/pour to septic tank, flush/pour to unknown place, pit latrine with slab, ventilated improved pit latrine, composting toilet. Unimproved: pit latrine without slab/open pit, no facilities or bush or field or surface water, hanging toilet/hanging latrine, flush/pour to open drains, unable to observe private latrine. | | | |

**Table S5.** Associations between socioeconomic variables and trachomatous inflammation—follicular (TF) among children aged 1-9 years in Alto Amazonas, Peru.

| **Characteristic** | TF Prevalence | Prevalence Ratio (95%CI)^a^ | P-value |
| --- | --- | --- | --- |
| **Occupation** |  |  |  |
| Non-agriculture | 12 / 74 (16%) | Ref |  |
| Agriculture | 172 / 557 (31%) | 1.50 (0.85–2.64) | 0.32 |
| Non-remunerated | 16 / 36 (44%) | 1.48 (0.86–2.56) |  |
| **Hectares farmed** |  |  |  |
| 0 | 33 / 128 (26%) | Ref |  |
| 1 | 78 / 246 (32%) | 1.27 (0.93–1.73) | 0.03 |
| 2 | 65 / 230 (28%) | 1.30 (0.95–1.77) |  |
| ≥ 3 | 24 / 63 (38%) | 1.96 (1.25–3.06) |  |
| **Chickens** |  |  |  |
| No | 15 / 78 (19%) | Ref |  |
| Yes | 185 / 589 (31%) | 1.58 (1.18–2.11) | 0.002 |
| **Pigs** |  |  |  |
| No | 187 / 631 (30%) | Ref |  |
| Yes | 13 / 36 (36%) | 1.28 (0.72–2.26) | 0.40 |
| **Cows** |  |  |  |
| No | 193 / 648 (30%) | Ref |  |
| Yes | 7 / 19 (37%) | 1.20 (0.59–2.48) | 0.61 |
| **Number of rooms** |  |  |  |
| One | 29 / 70 (41%) | 0.99 (0.82–1.20) | 0.94 |
| Two or more | 171 / 597 (29%) | Ref |  |
| **Floor type** |  |  |  |
| Cement / wood | 81 / 377 (21%) | Ref |  |
| Dirt | 119 / 290 (41%) | 1.22 (0.86–1.71) | 0.27 |
| **Window(s)** |  |  |  |
| Yes | 38 / 186 (20%) | Ref |  |
| No | 162 / 481 (34%) | 1.22 (0.90–1.67) | 0.21 |
| **Cooking fuel** |  |  |  |
| Electricity / natural gas | 0 / 10 (0%) | Ref |  |
| Wood | 200 / 657 (30%) | 8.49 (0.47–153)^b^ | 0.15 |
| **Dresser** |  |  |  |
| Yes | 1 / 20 (5%) | Ref |  |
| No | 199 / 647 (31%) | 4.34 (0.52–36.0) | 0.17 |
| **Mobile phone** |  |  |  |
| Yes | 16 / 80 (20%) | Ref |  |
| No | 184 / 587 (31%) | 1.09 (0.56–2.13) | 0.80 |
| **Radio** |  |  |  |
| Yes | 48 / 163 (29%) | Ref |  |
| No | 152 / 504 (30%) | 1.01 (0.79–1.30) | 0.92 |
| **Hammock** |  |  |  |
| Yes | 136 / 436 (31%) | Ref |  |
| No | 64 / 231 (28%) | 0.84 (0.62–1.12) | 0.23 |
| ^a^ Adjusted for age and district.  ^b^ Firth logistic regression, ignoring community-level clustering. | | | |

# **Figure S1.** Directed acyclic graph depicting putative causal pathway between poverty and trachoma.

**Figure S2.** Visualization of principal components analysis for hygiene factors (left column) and wealth factors (right column), including plots of the percentage contribution of each variable to each component (A, D) and plots of the first vs second principal component for (i) individual observations (B and E, colored by trachoma status) and (ii) for each variable (C and F, colored by quality of representation of each variable, with higher square cosine [cos2] values indicating greater alignment with the first two components.

**Figure S3.** Relationship between wealth and hygiene indices with trachomatous inflammation—follicular (TF). Loess curves depict the relationship of the wealth index (left) and hygiene index (right)—as calculated from a principal components analysis—and the prevalence of TF.
